# Molecular Characterization of Resistance to Soybean Rust (*Phakopsora pachyrhizi* Syd. & Syd.) in Soybean Cultivar DT 2000 (PI 635999)

**DOI:** 10.1371/journal.pone.0164493

**Published:** 2016-12-09

**Authors:** Tri D. Vuong, David R. Walker, Binh T. Nguyen, Tuyet T. Nguyen, Hoan X. Dinh, David L. Hyten, Perry B. Cregan, David A. Sleper, Jeong D. Lee, James G. Shannon, Henry T. Nguyen

**Affiliations:** 1 Division of Plant Sciences, University of Missouri, Columbia, Missouri, United States of America; 2 Soybean/Maize Germplasm, Pathology, and Genetics Research Unit, USDA-ARS, and Department of Crop Sciences, University of Illinois, Urbana, Illinois,United States of America; 3 Plant Protection Research Institute (PPRI), Ha Noi, Vietnam; 4 Soybean Genomics and Improvement Laboratory, USDA-ARS, Beltsville, Maryland, United States of America; 5 Division of Plant Sciences, University of Missouri, Portageville, Missouri, United States of America; USDA-ARS Southern Regional Research Center, UNITED STATES

## Abstract

Resistance to soybean rust (SBR), caused by *Phakopsora pachyrhizi* Syd. & Syd., has been identified in many soybean germplasm accessions and is conferred by either dominant or recessive genes that have been mapped to six independent loci (*Rpp1 –Rpp6*), but No U.S. cultivars are resistant to SBR. The cultivar DT 2000 (PI 635999) has resistance to *P*. *pachyrhizi* isolates and field populations from the United States as well as Vietnam. A F_6:7_ recombinant inbred line (RIL) population derived from Williams 82 × DT 2000 was used to identify genomic regions associated with resistance to SBR in the field in Ha Noi, Vietnam, and in Quincy, Florida, in 2008. Bulked segregant analysis (BSA) was conducted using the soybean single nucleotide polymorphism (SNP) USLP 1.0 panel along with simple sequence repeat (SSR) markers to detect regions of the genome associated with resistance. BSA identified four BARC_SNP markers near the *Rpp3* locus on chromosome (Chr.) 6. Genetic analysis identified an additional genomic region around the *Rpp4* locus on Chr. 18 that was significantly associated with variation in the area under disease progress curve (AUDPC) values and sporulation in Vietnam. Molecular markers tightly linked to the DT 2000 resistance alleles on Chrs. 6 and 18 will be useful for marker-assisted selection and backcrossing in order to pyramid these genes with other available SBR resistance genes to develop new varieties with enhanced and durable resistance to SBR.

## Introduction

Soybean rust (SBR), caused by the biotrophic fungal pathogen *Phakopsora pachyrhizi* Syd. & Syd., is considered to be one of the most destructive foliar diseases of soybean [*Glycine max* (L.) Merr.]. The disease was first reported in Japan in 1902, and has been known to drastically reduce soybean yields in Australia, Asia, Africa, and South America [1]. In the United States, SBR was first reported in Hawaii in 1994 [2] and was first observed in the continental United States in 2004 [3]. Since then, SBR has occurred annually in southern states, and was observed as far north as Ontario, Canada in 2007 (*sbr*.*ipmpipe*.*org/cgi-bin/sbr/public*.*cgi*).

When environmental conditions are conducive to disease development, SBR has caused yield losses of 40 to 90% in some Asian countries [4,5] and from 30 to 75% in Brazil and Paraguay [6]. Applications of fungicides are widely used to manage this disease, but can be costly if multiple applications are needed. Thus, resistant varieties with greater yield stability are desirable for long-term rust management [7]. To accomplish this, sources of unique and diverse *Rpp* resistance genes need to be identified and utilized.

Genetic studies of resistance to SBR have identified dominant *Rpp* genes at six different loci. These were named *Rpp1* [8], *Rpp2*, *Rpp3* [9], *Rpp4* [10], *Rpp5* [11] and *Rpp6* [12]. Garcia et al. [11] also discovered a recessive resistance allele (*rpp5*) in PI 200456. Monteros et al. [13] mapped a resistance gene (designated *Rpp*?Hyuuga) at the *Rpp3* locus in the Japanese cultivar Hyuuga (PI 506764), and Kendrick et al. [14] later found a second resistance gene in Hyuuga at the *Rpp5* locus. Chakraborty et al. [15] identified *Rpp1-b*, a different dominant resistance allele at the *Rpp1* locus in PI 594538A, Ray et al. [16] discovered additional resistance alleles at the *Rpp1* locus in PI 587886 and PI 587880A, and Hossain et al. [17] also reported other alleles at the *Rpp1* locus in PI 594767A and PI 587905. Harris et al. [18] found that 52 out of 75 resistant PIs had a resistance gene mapping to the *Rpp3* locus. While the original *Rpp1* gene from PI 200492 has provided high levels of resistance to U.S. *P*. *pachyrhizi* populations in multiple years and locations, other resistance alleles like *Rpp1-b* that were effective against foreign isolates failed to protect plants from SBR in the southern United States [19]. In addition to *Rpp* resistance genes identified in cultivated soybean, resistance has also been found in the wild perennial species *Glycine tomentella* [20]. Singh et al. [21] demonstrated the potential to backcross resistance genes from *G*. *tomentella* into *G*. *max* genetic backgrounds.

As noted earlier, each of the known *Rpp* genes conditions resistance to some, but not all *P*. *pachyrhizi* isolates and populations. In incompatible interactions, soybean genotypes with *Rpp* genes typically develop reddish-brown (RB) lesions, which are considered indicative of incomplete resistance [22,23]. In contrast, soybean genotypes producing tan-colored (TAN) reactions characterized by abundant sporulation from uredinia are fully susceptible. Immune, or Type 0 reactions have also been observed when plants with the *Rpp1* or *Rpp6* genes are challenged with specific *P*. *pachyrhizi* isolates. However, single-gene resistance to SBR in soybean has not been durable because *P*. *pachyrhizi* exhibits considerable variation in virulence among isolates and field populations, and certain strains have evolved the ability to overcome single-gene resistance that had previously been effective [7,16,24,25,26]. Thus, the identification of new SBR resistance sources with novel genes and different resistance mechanisms is important for the future development of resistant cultivars.

To identify new sources of resistance, Miles et al. [27] screened 16,595 accessions from the USDA-ARS Soybean Germplasm Collection for resistance to a mixture of four *P*. *pachyrhizi* isolates at the USDA-ARS Foreign Disease-Weed Science Research Unit (FDWSRU), at Fort Detrick, MD, and identified 805 accessions with low severity and/or RB lesions. Subsets of these accessions have subsequently been evaluated under field conditions in Nigeria, Paraguay, Vietnam and the United States [19,28–32]. In Vietnam, Pham et al. [30] reported that cultivar DT 2000, a local resistant accession, consistently developed RB lesions and had a low area under disease progress curve (AUDPC). These results were in agreement with an earlier study at the FDWSRU, in which DT 2000 was challenged with ten different rust isolates [29]. In field assays conducted in the southeastern USA between 2006 and 2013, DT 2000 was highly resistant in some year-location environments, but less resistant in others [19,32]. The objectives of the present study were to identify and characterize genomic locations or gene(s) associated with resistance to SBR in advanced inbred lines derived from DT 2000 under field conditions in Ha Noi, Vietnam, and Quincy, Florida, USA.

## Materials and Methods

### Plant materials

DT 2000 originated as a selection from the breeding line GC 00138–29, which was developed by the Asian Vegetable Research and Development Center (AVRDC) in Taiwan [33]. DT 2000 was resistant to soybean rust in net-house and field evaluations in Vietnam (Nguyen BT, unpublished data). Resistance of this cultivar to various *P*. *pachyrhizi* isolates from several countries has also been confirmed in greenhouse and field studies [30]. It was added to the USDA Soybean Germplasm Collection in 2004 as PI 635999 in the Germplasm Resources Information Network (GRIN, http://www.ars-grin.gov/npgs).

A genetic mapping population was developed from a cross of DT 2000 and the SBR-susceptible cultivar Williams 82 [34] at the University of Missouri-Columbia, MO, with DT 2000 as the paternal parent. Verified F_1_ plants were grown to produce F_2_ seeds in a winter nursery in Costa Rica. Over 250 individual F_2_ progeny from the population were advanced to develop recombinant inbred lines (RILs) using the single-seed descent (SSD) method. Two hundred and fifty F_6:7_ RILs and two parent lines were subsequently genotyped with DNA molecular markers and were phenotyped in the field for genetic characterization of SBR resistance.

### SBR reaction assays

Field experiments were conducted in 2008 at the Plant Protection Research Institute (PPRI) in Ha Noi, Vietnam, and at the University of Florida’s North Florida Research and Education Center (NFREC) in Quincy, Florida, USA. At the PPRI, the experimental design was a randomized complete block (RCB) with three replications. Twenty seeds of each entry were planted in 1-m single-row plots at a spacing of 60 cm x 5 cm. Five normal plants were used for disease ratings. A local susceptible soybean cultivar was grown in border rows as a source of inoculum. RILs and the parents were inoculated at the V6 (late vegetative) and R1 (early flowering) developmental stages [35] with a urediniospore suspension (5 × 10^4^ urediniospores/ml) of a local unpurified isolate of *P*. *pachyrhizi*. After inoculation the plots were thoroughly irrigated and covered with plastic sheets to maintain humidity for 12–16 h. Humidity was checked the following morning prior to removing the plastic sheets. For disease assessments, lesion types were recorded as being either RB, with little or no sporulation; TAN, with abundant sporulation; or a mixture of the two reaction types (Mixed) when most plants were at the R5 growth stage. In addition, disease severity was also assessed as percentage of symptomatic leaf area at the R3, R4, R5, and R6 growth stages on five plants at three leaf positions. An overall AUDPC measurement [36] was then calculated for each RIL and those values were used for further analysis. At the NFREC in Florida, the RIL population was grown in a completely randomized design (CRD. Seed of each entry was planted in single-row plots. Growing plants were naturally infected with urediniospores likely to have come from a susceptible local cultivar grown adjacent to the experiment plots. Lesion types (RB, TAN, or Mixed) were recorded using five to six normal plants in each plot. Disease severity was visually rated at the R5 growth stage using a rating scale of 1 (no visible lesions) to 5 (dense lesions), as previously described [32]. Sporulation levels of RILs and the parents were rated as previously described [29].

### DNA extraction and molecular marker analysis

Genomic DNA samples were isolated from young leaf tissue of the F_6:7_ RILs using the automated Autogen 960 system and the CTAB protocol of the manufacturer (AutoGen Inc., Holliston, MA, USA), with the minor modifications described by Vuong et al. [37,38].

Simple sequence repeat (SSR) analysis was also performed as described by Vuong et al. [37,38]. For single nucleotide polymorphism (SNP) genotyping, the Universal Soy Linkage Panel 1.0 (USLP 1.0), which is designed to query 1,536 SNP loci, was used with the Illumina GoldenGate assay [39], as described by Hyten et al. [40]. Allele determination for each SNP locus was subsequently performed using the intensity data imported into BeadStudio 3.0 software (Illumina, San Diego, CA, USA). The clusters of homozygous and heterozygous genotypes for each SNP were manually checked for genetic polymorphisms. Polymorphic SNP marker data were integrated with the SSR marker data to construct a genetic linkage map, as previously described by Vuong et al. [37].

### Bulked segregant analysis (BSA)

Bulked segregant analysis [41] was conducted using two DNA bulks that were assembled based on the leaf lesion types recorded in the field to test a model for inheritance. Briefly, 10–12 RILs that consistently developed either RB or TAN lesions among all replications and locations were selected. Normalized DNA samples of the lines with the same lesion type were then bulked. DNA samples from the two parental lines and the two DNA bulks were then genotyped using the USLP 1.0. The clusters of homozygous and heterozygous genotypes for each SNP were analyzed using BeadStudio 3.0 software (Illumina, San Diego, CA, USA) as described above.

### Selective genotyping

Selective genotyping [42] was carried out to assess partial resistance to SBR in DT 2000. Two sets of 10–12 RILs with highest and lowest AUDPC values were selected from the F_6:7_ RIL population evaluated at the PRRI in Vietnam. Normalized DNA samples of these lines were individually genotyped using the USLP 1.0 array to identify different genomic regions potentially associated with SBR resistance. Functions for selective genotyping analysis in the program MapQTL 5.0 were implemented for the SNP marker data collected in the study. Cluster analysis of allele calls was conducted similarly to the procedure described above.

### Data analysis

The AUDPC values, disease severity, and sporulation were tested for normality using the PROC UNIVARIATE procedure of SAS 9.1 (SAS Institute, Cary, NY, USA). The Shapiro-Wilk (*w*) statistic, skewness, and kurtosis were estimated to test the null hypothesis that these data were normally distributed [43].

A genetic linkage map was constructed using the program JoinMap 3.0 [44]. A likelihood of odds (LOD) threshold score of 3.0 and a maximum genetic distance of 50 cM were used for the initial linkage grouping of markers. For QTL mapping, a comprehensive analysis approach was performed using the program MapQTL 5.0 [44] to detect and map genomic regions significantly associated with SBR resistance, as previously described by Vuong et al. [37,38]. A genome-wide permutation test with 1000 random permutations was conducted on the genotypic and phenotypic data sets to obtain an empirically derived P ≤ 0.05 significance threshold for determining which LOD score peaks indicated the likely presence of a locus associated with variation in the phenotypic data. Epistatic interactions between QTL were predicted using the program QTLNetwork 2.0 [45] with a mixed-model. Significance levels for the genome scans for candidate intervals, QTL detection and effects were set at 0.05, 0.001, and 0.001, respectively. The linkage groups with LOD plots were subsequently created using the MapChart 2.2 program [46] based on the output from the programs JoinMap 3.0 and MapQTL 5.0 [44].

## Results

### Lesion type segregation

Results of field experiments conducted at the PPRI in Ha Noi, Vietnam and at the NFREC in Quincy, Florida, USA to evaluate resistance to SBR based on lesion types of each inbred line are presented in Table 1. Besides the TAN and RB lesion types, which have been commonly reported in previous genetic studies of reactions to SBR, both RB and TAN lesions were sometimes observed on the same RIL. These RILs were recorded as having a Mixed reaction. Among these types, it was noted that TAN reactions were predominant on 72% of the RILs grown at the PPRI and on 65% of those grown at the NFREC. The occurrence of Mixed lesions indicated that segregation for lesion type in this population differed from the expected 1:1 ratio normally expected for a single-gene inheritance pattern in an advanced inbred generation. The number of RILs with TAN lesions was greater than the number of lines with RB lesions, suggesting the possibility that the SBR resistance in DT 2000 might be controlled by more than one gene. In a two-gene segregation model, approximately one-fourth of the RILs should be homozygous for both resistance genes, one-fourth homozygous for both susceptible genes, and one-half homozygous for one or the other resistance gene. The observed segregation ratio could have also resulted at least partly from heterogeneous *P*. *pachyrhizi* populations/inoculum containing at least one pathotype that was able to overcome the resistance gene(s) in DT 2000.

**Table 1:** Reactions to soybean rust observed on F_6:7_ recombinant inbred lines (RILs) derived from a Williams 82 × DT 2000 cross and the parental lines in the field at the Plant Protection Research Institute (PPRI), Ha Noi, Vietnam, and at the North Florida Research and Education Center (NFREC), Quincy, Florida, USA, in 2008. Tan-colored (TAN) reactions indicate susceptibility and reddish-brown (RB) lesions indicate resistance. Mixed reactions might have resulted from the presence of multiple fungal pathotypes, residual heterozygosity, or a combination of these factors. Most RILs showed the similar reactions to rust at both locations.

Location	Number of F_6:7_ RILs observed			
	TAN	Mixed	RB	Total
PPRI, Ha Noi	180	21	50	251
	*(72%)*	*(8%)*	*(20%)*	
NFREC, Florida	160	38	48	246
	*(65%)*	*(15%)*	*(20%)*	

### Qualitatively assessed resistance and bulked segregant analysis (BSA)

Analysis of the lesion type data for the parental lines and the two DNA bulks showed that the genotypes at four linked BARC_SNP markers on chromosome (Chr.) 6 (corresponding to linkage group C2) were associated with resistance (Table 2). These SNP markers are located approximately 103 to 107 cM from the end of Chr. 6 based on the consensus linkage map [40]. This indicates the likely presence of a resistance gene at or near the *Rpp3* locus as previously mapped [47]. Markers BARC-023203-03824 and BARC-061709-17355 were mapped [40] to locations at 106.4 and 106.5 cM from the end of Chr. 6, respectively, and are tightly linked to the Satt079 and Satt460 SSR markers that were reported to be close to the *Rpp3* locus [47]. The two other SNPs, BARC-024739-05617 and BARC-051071-10973, mapped at 107.0 and 103.1 cM, putting them approximately 0.5 and 3.3 cM, respectively, from the estimated position of the *Rpp3* locus (Table 2).

**Table 2:** Single nucleotide polymorphism markers detected on the chromosome 6, co-segregating with the resistant and susceptible parental lines, DT 2000 and Williams 82, respectively, using bulked segregant analysis of recombinant inbred lines developed from a Williams 82 × DT 2000 cross.

BARC_SNP	DT 2000†	Williams 82†	R bulk†	S bulk†	Chr.‡	Genetic position (cM)‡	Associated SSR markers‡
BARC-051071-10973	BB	AA	BB	AA	6	103.1	Satt319, Satt489
BARC-023203-03824	BB	AA	BB	AA	6	106.4	Satt079, Satt460
BARC-061709-17355	BB	AA	BB	AA	6	106.5	Satt079, Satt460
BARC-024739-05617	BB	AA	BB	AA	6	107.0	Sat_238, Sat_263

^†^: Allele calls of each genotype were from the cluster analysis output of the BeadStudio program.

^‡^: Chromosome, genetic position, and associated SSR markers were accessed from the integrated soybean genetic linkage map [40] to localize a potential SBR resistance gene(s).

### Genomic regions associated with quantitative and semi-quantitative disease ratings

#### Phenotypic variation

**Fig 1:**
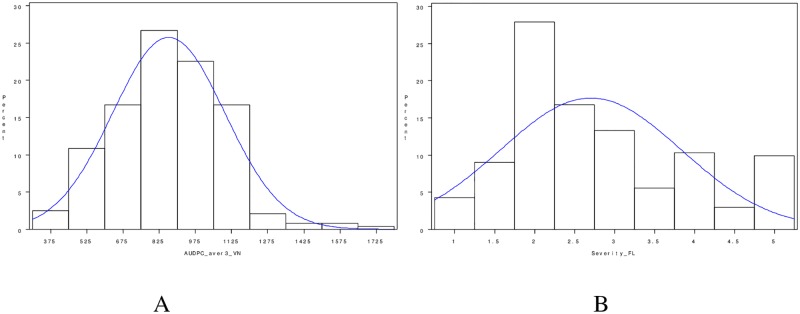
Distribution of disease resistance assessments of the mapping population evaluated at two field locations. (A) Area under disease progress curve (AUDPC) calculated for the evaluation at the PPRI, Ha Noi, Vietnam. (B) Disease severity calculated for the evaluation at the North Florida Research and Education Center (NFREC), Quincy, Florida, USA.

The distribution plots of AUDPC values and disease severity displayed continuous variation, and no discrete resistant and susceptible reaction classes were evident among the RILs evaluated (Fig 1). Normality tests using the Shapiro-Wilk (*w*) statistic, skewness, and kurtosis values indicated that only AUDPC values from the field evaluation in Ha Noi, Vietnam, were normally distributed, with a *w* value of 0.98 (*Pr*. value > 0.15). In contrast, disease severity and sporulation scored (data not shown) in the field evaluation at the NFREC in Florida were not normally distributed. The distributions of these two traits were slightly skewed toward the lower ratings for severity and sporulation (Table 3), but this problem would have been mitigated through the use of the genome-wide permutation tests used to calculate empirical significance thresholds for LOD score significance, together with CIM and cofactor marker selection as we have performed.

**Table 3:** Summary of soybean rust statistics for area under disease progress curve (AUDPC) data for soybean rust, disease severity, and sporulation (%) on parental lines and 240 F_6:7_ recombinant inbred lines derived from a Williams 82 × DT 2000 cross. The statistic Shapiro-Wilk (*w*), skewness, and kurtosis, were estimated to test the normality of the disease assessments.

Location	Disease assessment	Parent		F_6:7_ RIL			Shapiro-Wilk (*w*)	Skewness	Kurtosis
		Williams 82	DT 2000	Mean	Min	Max			
Ha Noi, Vietnam	AUDPC	1277.5	397.5	913.8	309.8	2113.0	0.98	0.4	0.9
	Sporulation	0.9	0.4	0.9	0.3	1.0	0.79	-1.2	0.7
Quincy, FL, USA	Severity	5.0	0.9	2.7	0.8	5.0	0.81	0.6	-0.5
	Sporulation	5.0	1.0	2.8	1.0	5.0	0.79	0.1	-1.7

#### Genetic linkage analysis

The entire F_6:7_ RIL mapping population was genotyped using the USLP 1.0 panel [40]. Of the 1,536 SNP markers in the panel, 484 (31.5%) were found to be polymorphic between the parents and were subsequently employed for genetic mapping. Besides the BARC SNPs, a subset of 25 existing SSR markers, which had been mapped and associated with the two genomic regions newly identified on Chrs. 6 (LG C2) and 18 (LG G) using the selective genotyping method (see the next section), were also tested and integrated into genetic linkage analysis. Altogether, a total of 504 SSR and SNP markers were successfully mapped on the 20 soybean chromosomes. The resulting map spanned approximately 2,250 cM, with an average genetic distance between markers of 2.5 cM (data not shown), but due to lack of polymorphic genetic markers in certain chromosomal regions, such as on Chr. 12 (LG H), some linkage fragments remained unmerged. Overall, marker orders in our molecular genetic map were consistent with those of the soybean composite linkage map [40], except for slight marker order rearrangements in a few chromosomal regions.

#### Selective genotyping

**Fig 2:**
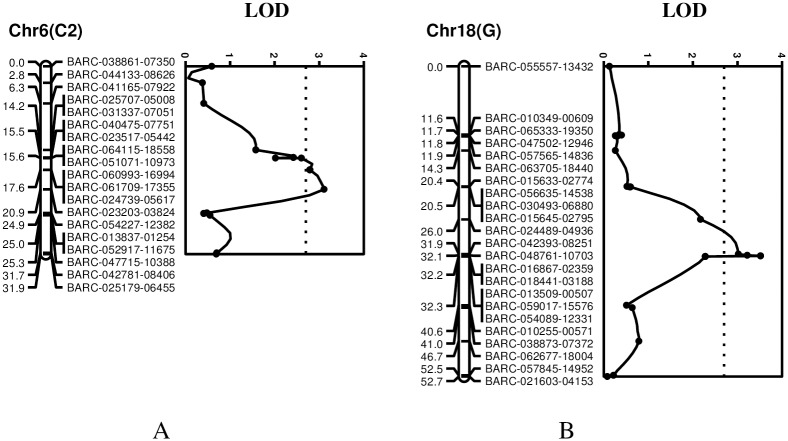
Genomic regions and BARC_SNP markers significantly associated with resistance to SBR were detected using the selective genotyping method in the Williams 82 × DT 2000 population. (A) A genomic region was mapped to Chr. 6 and closely linked to the *Rpp3* locus. (B) A second genomic region was mapped to Chr. 18 and closely linked to the *Rpp4* locus.

Rust resistance inherited from DT 2000 was assessed using AUDPC values from the experiment conducted at the PPRI. Selective genotyping was performed to identify genomic regions that were potentially associated with SBR resistance measured as phenotypic variation for AUDPC and disease severity. At the LOD significance threshold of 2.7 calculated using permutation tests, two chromosomal regions were found to be associated with variation in the AUDPC estimates (Fig 2). One of these, which was also detected using the BSA method, consistently mapped to Chr. 6 (LG C2) and a second genomic region association with variation in AUDPC values mapped to Chr. 18 (LG G) (Fig 2). The genomic region on Chr. 6 was a marker interval of 4.4 cM (Fig 2). Among several BARC SNPs associated with this region, it was noted that four markers previously identified using BSA were also closely associated with this genomic region. These included BARC-023203-03824, which was mapped at 20.9 cM and had a peak LOD score of 3.2 (Table 2). The favorable allele, which had an additive effect of 76.0 and explained 11% of the phenotypic variation, was inherited from DT 2000. The second region on Chr. 18 was mapped within a marker interval of 4.2 cM. Several SNP markers were closely associated with this genomic region (Fig 2). Among these, both BARC-016867-02359 and BARC-018441-03188 were mapped close to the peak at 32.2 cM with a LOD score of 3.5. DT 2000 had a favorable allele conferring greater resistance, with an additive effect of 79.2. The genotype at that locus accounted for about 11% of the variation in AUDPC values. According to the SSR- and SNP-based integrated genetic linkage map [40], these genomic regions corresponded to the locations of the *Rpp3* and *Rpp4* loci on Chrs. 6 and 18, respectively.

#### Genomic loci associated with resistance to SBR

Genetic mapping of resistance to SBR utilized different disease assessments obtained at two field locations in 2008. Among these, AUDPC values and sporulation (%) were estimated in a study at the PPRI in Ha Noi, while severity and sporulation rated on semi-quantitative scales were estimated in a study at the NFREC in Florida. Genome-wide permutation tests performed for pathogeneity assessments determined an average significant LOD threshold value of 3.4 (*Pr* = 0.05) for declaring a significant QTL.

**Fig 3:**
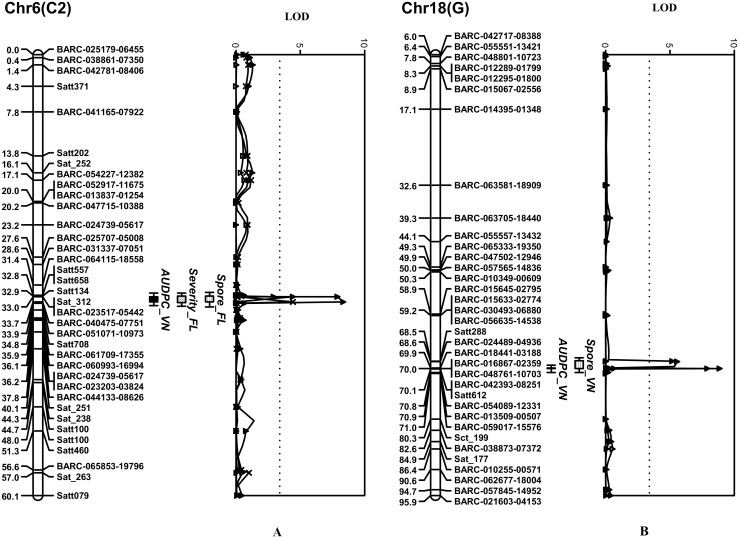
Genomic regions and SNP and SSR markers significantly associated with resistance to SBR were consistently detected using the composite interval mapping (CIM) method in the Williams 82 × DT 2000 population. (A) A genomic region was mapped to Chr. 6 and closely linked to the *Rpp3* locus. (B) A second genomic region was mapped to Chr. 18 and closely linked to the *Rpp4* locus.

In the test at the PPRI, the first resistance locus was mapped to Chr. 6. It had a maximum LOD score of 8.3 at the peak and explained 11.7% of the phenotypic variation observed (Table 4). Based on the 1-LOD confidence interval, the *Rpp* locus was located within a 0.8 cM genomic region flanked by BARC-023517-05442 and BARC-040475-07751, which mapped at 33.0 and 33.7 cM, respectively (Table 4, Fig 3). The second locus detected using the AUDPC data was mapped to Chr. 18. This locus had a maximum LOD score of 8.8 at the peak, and accounted for 12.5% of the phenotypic variation (Table 4). This locus was located within a 0.1 cM genomic interval flanked by the markers BARC-016867-02359 and BARC-048761-10703. The favorable alleles on Chr. 6 and 18 genomic loci were both inherited from the resistant parent, DT 2000, and had additive effects on reducing the AUDPC values (Table 4). An epistasis analysis that was subsequently performed to evaluate the interaction between these loci did not detect an epistatic interaction between the two loci mapped on Chrs. 6 and 18.

**Table 4:** Molecular marker intervals, LOD scores, *R*^2^ values, and additive effects (A) of genomic regions associated with resistance to soybean rust were calculated from composite interval mapping (CIM) analysis using the program MapQTL 5.0. These regions were mapped on Chrs. 6 and 18 using a F_6:7_ mapping population derived from a Williams 82 × DT 2000 cross.

Location	Disease assessment	Chr. 6 (C2)				Chr. 18 (G)			
		Marker interval	LOD	*R*^*2*^	*A*	Marker interval	LOD	*R*^*2*^	*A*
Ha Noi, Vietnam	AUDPC	BARC-023517-05442_BARC-040475-07751	8.3	11.7	110	BARC-016867-02359_BARC-048761-10703	8.8	12.5	116
	Sporulation	-	-	-	-	Satt288_BARC-024489-04936	5.6	9.6	0.02
Quincy, FL, USA	Severity	BARC-040475-07751_BARC-051071-10973	4.6	8.6	0.35	-	-	-	-
	Sporulation	Sat_312_BARC-203517-05442	4.2	8.4	0.52	-	-	-	-

Mapping using sporulation assessment data from Ha Noi detected one locus on Chr. 18 in a 0.1 cM genomic interval flanked by the SSR marker Satt288 and the SNP marker BARC-024489-04936, which were located at 68.4 and 68.5 cM, respectively (Fig 3). The maximum LOD score was 5.6, and the genotype at the marker loci explained 9.6% of the phenotypic variation in sporulation (Table 4). This locus was close to the interval detected on Chr. 18 using the AUDPC values (Fig 3). No significant peaks associated with variation in sporulation were identified on Chr. 6, despite the fact that the single marker-trait correlation analysis revealed significant correlation coefficients (data not shown).

Using data from the NFREC in Florida, one locus associated with variation in disease severity was detected which consistently mapped to the same genomic region of Chr. 6 as the locus detected using the AUDPC data from Vietnam (Fig 3). Two SNPs, BARC-023517-05442 and BARC-051071-10973, flank a genomic interval of 0.1 cM (Fig 3) with a LOD score of 4.6 at the peak, and explained 8.6% of phenotypic variation (Table 4). Similarly, a genomic region associated with sporulation intensity was also mapped to the same region of Chr. 6, where its estimated location overlapped with that of the region detected using the AUDPC values and the Chr. 6 locus associated with variation in severity (Fig 3). The LOD score, *R*^*2*^ value, additive effect, and flanking markers of this locus are shown in Table 4.

Similar to the allele effects of the putative loci on Chrs. 6 and 18 determined in the study at the PPRI, DT 2000 contributed the favorable alleles at the markers on Chr. 6 associated with lower SBR severity and sporulation. In contrast to the findings in the field experiment at the PPRI, neither of the genomic regions on Chr. 18 associated with severity or sporulation was detected using the field evaluation data from Florida.

## Discussion

In the RIL mapping population assayed for the present study, rust-infected plants in a majority of the lines developed the type of TAN lesions associated with susceptibility. In previous genetic studies of resistance to SBR, dominant (*Rpp*), recessive (*rpp*), and incompletely dominant resistance genes have been identified in crosses with various resistance sources [8–13,15,16,48]. The presence of mixed reactions was also reported in genetic populations derived from PI 200456 and PI 224270 [48]. In addition, inversion of dominance by a susceptibility allele has been reported in populations derived from the cross of some PIs crossed with different susceptible parents [49]. In our field evaluations in Ha Noi, the mapping population was inoculated with locally collected unpurified inoculum. The results (Table 1) showed a similar pattern to those from some previous reports [11,48]. It indicated that DT 2000 carries SBR resistance genes at more than one locus, rather than a single *Rpp* gene.

Although the *Rpp3* gene from PI 462312 and the *Rpp4* gene from PI 459025B, exhibit gene-for-gene interactions with different isolates of *P*. *pachyrhizi* and are considered to be major R genes, it should be borne in mind that *Rpp* genes seldom condition immunity to the SBR fungus. Incomplete resistance, combined with the possibility of differential interactions between the two *Rpp* genes and different pathotypes of the fungus present in the field, could account for why the *R*^2^ estimates of the portion of phenotypic variation explained by the genotypes at markers near the two *Rpp* loci shown in Table 4 are lower than what might be expected for a major R gene. At the time the field assays were conducted, we did not fully appreciate the amount of pathogenic variation that can exist in field populations of *P*. *pachyrhizi*, and believed that phenotypic reactions to field populations would provide robust rating data. Subsequent investigations of genetic and pathogenic variation in *P*. *pachyrhizi* populations have shown, however, that considerable variation can exist among and within fields [50–52]. The possibility that multiple pathotypes might have been present in the fields where the reaction evaluations were conducted, along with pathotype × host interactions, could therefore account in part for the continuous distribution of reaction data. Some portion of the observed phenotypic variation might also have been due to non-uniform infections, particularly when natural infection occurred from neighboring border rows. Despite these factors which likely contributed some experimental error to the ratings, the data were sufficiently accurate to allow the detection of markers tightly linked to two known *Rpp* genes.

Walker et al. [19,32] evaluated the resistance of DT 2000 to field populations of *P*. *pachyrhizi* in the southern United States in 2007 (three locations), 2008 (five locations), 2009 (five locations), 2011 (one location) and 2012 (two locations). In 2007 and 2008, DT 2000 and PI 462312 (*Rpp3*) appeared to have similar levels of resistance at most locations, with both showing more resistance than PI 459025B, the source of the *Rpp4* gene [32]. At Baton Rouge, LA, in 2008, however, SBR severity on DT 2000 was higher than that observed on some susceptible checks. In 2009, DT 2000 had low disease severity and low sporulation at locations in Alabama, Georgia, Florida and South Carolina, but was highly susceptible to the rust population in Bossier City, LA, as were the accessions with the *Rpp3* or *Rpp4* genes and numerous other historically resistant plant introductions. DT 2000 was also susceptible to a 2011 fungal population in northern Florida, but showed better resistance there and in Attapulgus, GA, in 2012. The resistance of DT 2000 also appeared to be superior to that conditioned by the *Rpp3* or *Rpp4* gene in 2012. Overall, the data indicated that the genes in DT 2000 should provide at least moderate protection against many *P*. *pachyrhizi* populations in the southern United States.

For a qualitatively inherited trait, BSA has been demonstrated to be an efficient approach to localize resistance gene(s) based on the genetic association of molecular markers and bulk phenotypes [41]. In our study, several SNP markers close to the *Rpp3* locus on Chr. 6 were identified using BSA (Table 2). An *Rpp* gene associated with lesion type was mapped to the genomic location of the *Rpp3* locus [47], but it is not yet known whether DT 2000 carries the same allele as PI 462312, the source of the original *Rpp3* gene. Other studies have shown that numerous SBR-resistant soybean germplasm accessions carry a resistance gene at the *Rpp3* locus [13,17,18,47].

Analysis of data from selected genotypes identified a second genomic location, where several associated SNP markers were detected and mapped on Chr. 18 (Fig 2) at a location that overlapped the *Rpp4* locus [53]. Because of the restrictions of the selective genotyping approach, only the interval mapping (IM) method was used [44]. Selective genotyping limits the advantages of the composite interval mapping (CIM) and cofactor selection methods, which often improve the precision genetic mapping. However, the detection of a second genomic location associated with SBR resistance indicated that DT 2000 has a unique SBR resistance that involves two genomic regions corresponding to the locations of the *Rpp3* and *Rpp4* loci.

Results of the selective genotyping analysis inspired us to try mapping the genes using phenotypic data from the entire RIL population evaluated at the two locations. Similar to the results of selective genotyping, two genomic locations significantly associated with variation in the AUDPC values, SBR severity, and sporulation from uredinia were detected and mapped on Chrs. 6 and 18 in the regions containing the *Rpp3* and *Rpp4* loci, respectively. Moreover, it allowed the 4.4-cM and 4.2-cM marker intervals identified by selective genotyping (Fig 2) to be narrowed to less than 0.1 cM (Fig 3). CIM also resulted in higher LOD scores and *R*^*2*^ values (Table 4). In addition to the BARC_SNPs, polymorphic SSR markers from the vicinities of the *Rpp3* and *Rpp4* loci were also included in the analysis. Genetic positions of these genetic markers were in an agreement with the consensus linkage map [54]. In particular, SSR markers in this study and their genetic positions on Chrs. 6 and 18 were consistent with the results of previous studies to map *Rpp* genes [13,17,47,53]. Because of the nature of the pathogenicity of soybean rust and the host resistance in diverse soybean germplasm, it is desirable to monitor disease progress over time [5,7]. Moreover, it has been recommended that the employment of new sources with partial resistance in soybean breeding programs could potentially overcome the ineffectiveness of race-specific monogenic resistance [7,55].

An earlier study [29] demonstrated that rust populations can be pathogenically diverse. The genotype at the locus on Chr. 6 was associated with SBR resistance in both locations based on significant associations with AUDPC values, SBR severity, and sporulation intensity (Fig 3). In contrast, the genotype at the locus on Chr. 18 was only associated with resistance measured by AUDPC values and sporulation intensity in Ha Noi, and was not detected using the data from Quincy, FL. It should be noted that across multiple locations and years in the southern USA, the original *Rpp4* gene from PI 459025B seldom provided very high levels of resistance to SBR, whereas the *Rpp3* gene from PI 462312 typically conditioned moderately high levels of resistance [19,32]. It is therefore possible that the allele at the *Rpp4* locus of DT 2000 was not detected using the data from Florida because that allele did not condition effective resistance against the local *P*. *pachyrhizi* population. Thus, further investigation is needed to determine whether the SBR resistance of DT 2000 is controlled by the same genes/alleles as those previously reported [13,17,18,47,53]. Moreover, it is worth noting that a number of the USDA germplasm accessions that have shown resistance to *P*. *pachyrhizi* field populations in the southern USA originated from northern Vietnam [19,32]. This appears to be more than coincidence, and may indicate some historical connection between the SBR populations that have become established in North America and the fungal populations in northern Vietnam.

In conclusion, the results of the present study indicated that the SBR resistance of DT 2000 was conditioned by genes/alleles at two genomic regions on Chrs. 6 and 18 that most likely correspond to the *Rpp3* and *Rpp4* loci. Under the field evaluations in Vietnam and the southern USA, the resistance genes inherited from DT 2000 significantly reduced AUDPC values, disease severity, and/or sporulation intensity. SSR and SNP markers tightly linked to these loci can be used for marker-assisted selection or backcrossing to pyramid the *Rpp* genes from DT 2000 with other available SBR resistance genes. In theory, this will provide more durable resistance against pathogenically complex pathogen populations. Because DT 2000 is a cultivar, there should be fewer problems with adverse effects on yield and other agronomically important traits resulting from linkage drag if DT 2000 is used as a source of resistance genes.
